# Editorial: Integrating genetics and proteomics for drug discovery

**DOI:** 10.3389/fgene.2026.1806492

**Published:** 2026-02-16

**Authors:** Karunakaran Kalesh, Liang Xue

**Affiliations:** 1 School of Health and Life Sciences, Teesside University, Middlesbrough, United Kingdom; 2 Research and Development, Pfizer Inc., Cambridge, MA, United States

**Keywords:** biomarker, co-localization, drug discovery, genetics, mendelian randomisation, protein quantitative trait loci (pQTL), proteogenomics, proteomics

## Why this Research Topic matters

Genetics has mapped thousands of robust, human-anchored associations with disease, yet many programmes still stall when attempting to move from “variant” to “valid target.” Integrating genetics with proteomics helps close this gap by testing whether genetic signals manifest as protein-level changes in the relevant tissues, pathways, and cell states. This integration strengthens causal inference (via pQTLs, colocalization, and Mendelian randomization), reveals drug-amenable biology such as isoforms, complexes, post-translational regulation, and yields biomarkers that reflect underling mechanism rather than superficial correlation (Sun et al., 2018; Giambartolomei et al., 2014). In short, it advances discovery from statistical association to experimentally tractable hypotheses.

Equally, it makes development more predictable. Protein-aware, genetics-informed strategies help anticipate on-target safety liabilities using human data. They also support the design of pharmacodynamic readouts that align with mechanism and enable patient stratification based on the biology a medicine truly modulates. As platforms expand—from bulk tissue-based and affinity-based assays to single-cell and spatial modalities—rigorous harmonization and transparent analytic pipelines become essential. These practices ensure that multi-omic findings generalize across cohorts and remain reliable for use in clinical trials.

Together, the five papers in this Research Topic demonstrate how integrating multi-omic data can (i) elevate genetically supported causal targets, (ii) reveal biomarker candidates for patient stratification and pharmacodynamic (PD) monitoring, and (iii) sharpen clinical trial design by embedding findings within a systems-level biological context.

## From associations to actionable biology

A central theme is the move beyond single-omic signals to convergent evidence across layers. Gong et al. conduct a systematic, tiered integration of genome-wide association studies (GWAS) with expression (eQTL), protein (pQTL), and metabolite (mQTL) quantitative trait loci to prioritize mitochondrial genes across prostatic diseases. They then test whether the observed effects are mediated through metabolites and immune traits. Their framework highlights **DCXR** (Tier 1) as a candidate for benign prostatic hyperplasia and nominates additional gene–disease links—such as **ELAC2** and **ACAT1**—that become tractable hypotheses for experimental perturbation and pharmacodynamic (PD) readouts in early development.

Jin et al. take a complementary, disease-centred route in hepatocellular carcinoma, aligning transcriptomics with proteomics from the Clinical Proteomic Tumour Analysis Consortium (CPTAC), copy-number variation (CNV) data, and tumour-microenvironment (TME) analyses. They show that several pseudouridine synthases - RNA-modifying enzymes such as **DKC1**, **PUS1**, and **PUS7** - are upregulated and have diagnostic and prognostic value. By linking these RNA-modification enzymes to pathway activity and immune context, they outline a mechanistic space where genetic or pharmacological modulation could intersect with biomarker-driven trials.

Jiang et al. explore lipid metabolism at the interface of tumour immunity, nominating **CPT2** (carnitine palmitoyltransferase 2) as a pan-cancer biomarker candidate linked to immune-cell infiltration and survival in patients receiving immunotherapy. Although primarily a computational analysis, their work argues for incorporating metabolic–immune axes such as fatty-acid oxidation (FAO) into proteomic panels used for patient stratification or pharmacodynamic (PD) monitoring in mechanism-anchored studies.

## Proteogenomics, platforms and practicality

Philips et al. review opportunities for predictive proteogenomics biomarkers of drug sensitivity in epithelial ovarian cancer, a setting where BRCA1/BRCA2 status and homologous recombination deficiency (HRD) are necessary but not sufficient for treatment decisions. They highlight use cases in which proteome-level pathway activity—such as DNA-repair programs or immune signatures—and target-adjacent proteins help explain why patients vary in their responses to poly(ADP-ribose) polymerase (PARP) inhibitors, anti-angiogenic agents, and antibody–drug conjugates (ADCs). For drug discovery, these insights support trial designs that include proteogenomics baselines (paired genomic and proteomic profiling before therapy) and on-treatment proteomics to confirm mechanism engagement and detect early resistance biology.

As single-cell technologies scale, the bibliometric analysis by Chen et al. highlights how rapidly increasing resolution and throughput are accompanied by mounting integration challenges. Their mapping of methodological hotspots underscores the need for rigorous cross-platform harmonization. This includes careful batch handling, the use of bridge samples to link datasets, and attention to epitope-altering variants that can distort affinity-based assays. It also highlights the importance of transparent analysis pipelines that explicitly connect cell-type-resolved expression to protein changes and clinical phenotypes. Together, these practices form the foundation for achieving reproducible causal inference in multi-omic studies.

## An emerging playbook for genetically-informed proteomics

Taken together, the Research Topic points to a pragmatic playbook:

1. Elevate causal targets with convergent instruments. Use cis-pQTLs and co-localization to raise the prior on true target-disease links; where feasible, triangulate with eQTLs, mQTLs and metabolite instruments. Gong et al.’s tiering provides a template for rank-ordering genes before expensive wet-lab validation.
2. Design biomarkers on the causal path. Prioritise proteins that either *mediate* genetic risk or report on target-pathway modulation. Jin et al. and Jiang et al. illustrate how pathway-anchored proteins (RNA-modification enzymes; fatty-acid oxidation components) can serve as diagnostic and prognostic markers and candidate PD readouts.
3. Exploit proteogenomics where DNA alone is silent. Isoform-specific or post-transcriptional effects often determine druggability and resistance. Philips et al. make the case for proteogenomics baselines and on-treatment sampling to capture pathway rewiring that genomics alone misses.
4. Plan for scale and heterogeneity. As Chen et al. show, single-cell and spatial methods are exploding; combining these with bulk and affinity proteomics will demand rigorous QC, common data models, and pre-registered analysis plans so findings generalise across cohorts and platforms.

## Outlook: from genetic clues to actionable studies

Begin with targets grounded in human genetics. Then verify—using the relevant tissues and cell types—that the intervention modulates the protein and perturbs the pathway as intended. Define the purpose of each biomarker upfront: prognostic (risk), predictive (who benefits), or pharmacodynamic (evidence of target engagement). Establish decision-enabling thresholds early in development to ensure biomarkers can guide actionable choices. (Nelson et al., 2015). By pairing causal genetics with proteomic readouts and practical measurement plans, we can move more quickly from association to action. This approach supports the selection of safer targets, the design of smarter trials, and the earlier reduction of development risk.
